# Bioactivity of Polyphenols: Preventive and Adjuvant Strategies toward Reducing Inflammatory Bowel Diseases—Promises, Perspectives, and Pitfalls

**DOI:** 10.1155/2016/9346470

**Published:** 2016-07-10

**Authors:** Anouk Kaulmann, Torsten Bohn

**Affiliations:** ^1^Luxembourg Institute of Science and Technology, Environmental Research and Innovation Department, 41 rue du Brill, 4422 Belvaux, Luxembourg; ^2^Luxembourg Institute of Health, Population Health Department, 1 A-B rue Thomas Edison, 1445 Strassen, Luxembourg

## Abstract

Inflammatory bowel diseases (IBDs) are characterized by autoimmune and inflammation-related complications of the large intestine (ulcerative colitis) and additional parts of the digestive tract (Crohn's disease). Complications include pain, diarrhoea, chronic inflammation, and cancer. IBD prevalence has increased during the past decades, especially in Westernized countries, being as high as 1%. As prognosis is poor and medication often ineffective or causing side effects, additional preventive/adjuvant strategies are sought. A possible approach is via diets rich in protective constituents. Polyphenols, the most abundant phytochemicals, have been associated with anti-inflammatory, antioxidant, immunomodulatory, and apoptotic properties. Locally reducing oxidative stress, they can further act on cellular targets, altering gene expression related to inflammation, including NF-*κ*B, Nrf-2, Jak/STAT, and MAPKs, suppressing downstream cytokine formation (e.g., IL-8, IL-1*β*, and TNF-*α*), and boosting the bodies' own antioxidant status (HO-1, SOD, and GPx). Moreover, they may promote, as prebiotics, healthy microbiota (e.g., Bifidobacteria,* Akkermansia*), short-chain fatty acid formation, and reduced gut permeability/improved tight junction stability. However, potential adverse effects such as acting as prooxidants, or perturbations of efflux transporters and phase I/II metabolizing enzymes, with increased uptake of undesired xenobiotics, should also be considered. In this review, we summarize current knowledge around preventive and arbitrary actions of polyphenols targeting IBD.

## 1. Introduction—Preventive Strategies for IBD

### 1.1. General Aspects

Inflammatory bowel diseases (IBDs) are on the rise. With annual incidence rate (newly diagnosed diseases) up to 1‰ and a prevalence of 1% in many developed countries [1], this complication is affecting considerably more people than in the past, for reasons unknown. Crohn's disease (CD) and ulcerative colitis (UC) are the main forms of the disease, with CD resulting in manifestations in the small and large intestine, while UC is confined to the colon. Typically, the disease manifests itself before 30 years of age, and most likely genetic predisposition followed by autoimmune reactions does play a role in their aetiology, though concrete reasons or triggers are not understood. Symptoms include diarrhoea, abdominal pain, cramping, fever, weight loss, wasting, internal bleeding, and ultimately cancer. In both diseases, the epithelial lining of the gut is in part destroyed, resulting in perturbed permeability of the mucosal barrier, malabsorption of nutrients, and absorption of compounds by-passing the enterocytes, causing, for example, allergic reactions, a circumstance often described as “leaky gut syndrome” [2, 3]. Many subjects present with low concentrations of essential micronutrients such as vitamins and minerals, especially zinc, iron, selenium, vitamin B12, and vitamin D [4–7], possibly (a) due to low dietary intake and avoidance of many food products expected to cause digestional discomfort, (b) due to diarrhoea, loss of blood, and malabsorption in the inflamed areas, and perhaps (c) due to enhanced metabolism/turnover of some of these essential micronutrients (such as antioxidant vitamins).

### 1.2. Pathophysiological Description of Condition

Several differences between CD and UC exist. First, while CD can affect both the small and the large intestine (in addition to mouth and stomach), UC is limited to the colon. However, most typically, CD affects the lower parts of the small intestine (distant ileum) and the upper parts of the colon. Second, another distinction is that while CD may affect the entire gut wall, UC typically affects only the inner lining (mucosa, submucosa) of the epithelium. Third, CD may affect certain areas and leave intermittent parts (“skip areas”) intact, which is not observed in UC [8]. In CD, crypt inflammation and abscesses may turn into mucosal oedema, thickening of the bowel wall, and fibrosis and fistula development (extending to other organs such as the bladder), among others. UC typically starts with the rectum, mucosal ulcers are common, and fistulas and abscesses are absent.

Why the epithelial lining and additional adjacent tissues are inflicted is not entirely understood, but autoimmune reactions appear to be involved [9], characterized by local spots of increased inflammation, including infiltration of immune cells. Several cell types are involved in this response, including absorptive enterocytes, mucus-producing goblet cells, enteroendocrine cells (secreting hormones such as cholecystokinin), paneth cells (required for bacteria defence), microfold cells (M-cells, taking up antigens via endocytosis), and additional infiltrating cells of the immune system, such as neutrophils. It has been reported that secondary lymphoid tissues, for example, Peyer's patches, and tertiary lymphoid tissues can respond to antigen stimuli, releasing cytokines and antibodies (IgA, [10]). Cell surface receptors (toll-like receptors (TLRs) and nod-like receptors (NLRs)), located on many cells of the immune system, infiltrating to the diseased tissue, may sense pathogen associated molecules. Herein lies an important interaction with the gut microbiota, as certain bacteria such as Bacteroides can interact with, for example, T regulatory cells and macrophages, stimulating anti-inflammatory IL-10 production, while other bacteria may induce T-helper- (Th-) 17 cells, fostering inflammation.

**Figure 1:**
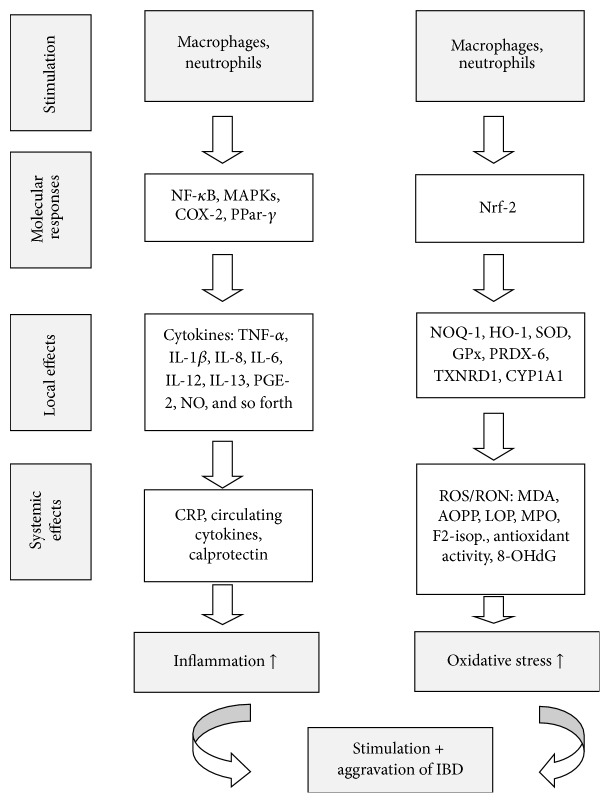
Factors involved in the origin and progression of IBD, via inflammation and oxidative stress. For abbreviations see footnote of Table 3.

**Figure 2:**
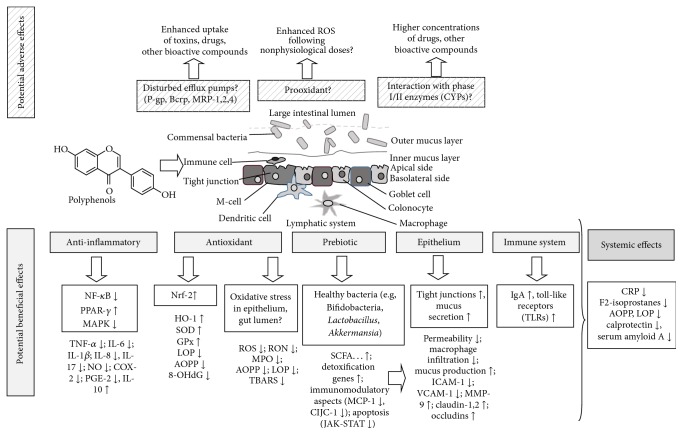
Summary of mechanisms via which polyphenols may positively or negatively influence the development of IBD. For abbreviations see footnote of Table 3.

However, this process is characterized not only by local inflammation (Figure 1), but by a systemic low chronic inflammation, with increased concentrations of circulating cytokines, especially IL-8, TNF-*α*, and IL-1*β* [11], and other general markers of inflammation such as C-reactive proteins (CRP) [12]. It is believed that IBD is mostly triggered and aggravated by TNF-*α* released from infiltrating immune cells (macrophages), followed by increased concentrations of the cytokines IL-6 and IL-1*β* [13] and possibly IL-12 (especially for CD) and IL-13 (especially UC) [14], and reduced concentration of the anti-inflammatory cytokines IL-10 and IL-4 [15]. Immune cells (neutrophils and macrophages) also produce a number of reactive oxygen species (ROS) in order to trigger further inflammation, resulting in reduced plasma antioxidant activity. This typically goes along with increased levels of myeloperoxidase (MPO, producing ROS from hydrogen peroxide [16]), causing enhanced formation of lipid oxidized products (LOP) such as malondialdehyde (MDA) and advanced oxidized protein products (AOPP, Figure 2). This in turn may be accompanied with increased levels of markers of (nonenzymatic) oxidative stress, such as F2-isoprostanes [17].

Despite the fact that the precise reasons for the increased prevalence of IBD are still controversially discussed, certain environmental aspects appear to play a role (Table 1), such as smoking, hygiene, certain microorganisms, use of oral contraceptives (OCPs), nonsteroidal anti-inflammatories (NSAIDs), antibiotics, appendectomy, breastfeeding, ambient air pollution [18], the gut microbiota [19], and certain diet related habits, such as high fat consumption, consumption of refined sugars, and low vitamin D intake, at least according to some studies [18]. A genetic predisposition has also been reported [20, 21]. Certain mutations have been revealed, such as (for CD subjects) the gene encoding for NOD2 (nucleotide-binding oligomerization domain 2) [14]. A similarity between UC and CD with the aetiology of celiac disease (CeD) also exists, with the main difference that people may stay asymptomatic with CeD, as long as the known antigen triggering the disease, that is, gluten, is avoided [22].

### 1.3. Strategies for Ameliorating IBD

Strategies to resolve complications have been including the administration of nonsteroidal anti-inflammatory drugs [23], steroid anti-inflammatories, typically targeting TNF-*α* reduction [24] or, for the worst cases, surgical removal of the inflammatory afflicted areas. A standard therapy is the administration of 5-aminosalicylic acid (a nonsteroidal anti-inflammatory drug), which reduces (via cyclooxygenase-2 (Cox-2)) the formation of leukotrienes and prostaglandins, focussing on blocking inflammatory processes, that is, their mediators [8]. Nevertheless, drugs usually do not result in complete cure, and relapse rates, even after operation, remain relatively high.

As a consequence, preventive strategies appear as a prudent approach to avoid IBD, which is complicated by the fact that the causes of IBD are not fully understood. Nevertheless, a diet rich in fruits and vegetables has been shown to be able to reduce the incidence/prevalence of IBD [25], perhaps due to positive effects of dietary fiber [26]. Dietary fiber promotes the production of short chain fatty acids (SCFAs) in the colon, and these have been reported to possess anti-inflammatory and immunomodulatory effects. Fibre would also reduce colonic pH, inhibiting the growth of potential pathogenic microbes. This has been suggested especially for selected dietary fibers such as prebiotics [27]. Consequently, also probiotics have been promoted to reduce IBD symptoms; for example, some positive effects were seen for probiotics and UC [28], maintaining remission, while effects on CD have not been shown so far.

An additional class of compounds, often affiliated in the fruit and vegetable matrix with dietary fiber, which has recently attracted much attention, are polyphenols, the most abundant secondary plant compounds or phytochemicals. Polyphenols, sometimes also termed phenolics, constitute a broad class of compounds, comprising over 7000 compounds. They can further be subdivided into flavonoids (such as isoflavonoids and anthocyanins) and nonflavonoids (e.g., stilbenes, phenolic acids, coumarins, and tannins). Their concentration in some fruits and vegetables (Table 2) can be as high as several 100 mg/100 g [29], and their per capita intake typically ranges around 1 g/d [30]. It is also important to consider that the majority of polyphenols (possibly as high as 90–95%) is not absorbed but reaches the colon [31] and is thus available as a substrate for fermentation. A review on the most prominent polyphenol containing food items was published by Pérez-Jiménez et al. [32]. In the following, we try to summarize the various pathways via which polyphenols could act on the development of IBD. More specifically, aspects related to

- direct and indirect antioxidant,
- anti-inflammatory,
- gut microflora,
- other properties with respect to epithelium protection, such as their influence on tight junctions,

are discussed, as these appear to constitute major mechanisms of action of polyphenols with respect to potential health benefits targeting IBD prevention or amelioration. In addition, potential negative effects of polyphenols are also briefly discussed. For this purpose, searches in PubMed for all years of English literature were carried out, employing the search terms “(polyphenol? or phenolic? or flavonoid) and (IBD or inflammatory bowel disease or Crohn's disease or ulcerative colitis)”.

## 2. Insights from Human Trials

### 2.1. Epidemiological Insights

A limited number of epidemiological trials have suggested a positive association between fruit and vegetable consumption and IBD. For example, in a prospective cohort study with over 170.000 women participating in the Nurses' Health Study [115], subjects consuming the most dietary fiber had a 40% lower risk of developing CD (OR 0.59, 95 CI 0.39–0.90). Positive influences on the gut microbiota and the aryl-hydrocarbon receptor (AhR), mediating protection against xenobiotics, were discussed. Interestingly, fiber from fruits showed greatest effects, while fiber from cereals and whole grains appeared not to alter the risk, which may have been due to additional effects of polyphenols, as extractable polyphenols are especially associated with various fruits. It should also be noted that fiber intake did not appear to influence UC in this study. Similarly, a systematic review of the literature suggested that the intake of fiber and high fruit intake was associated with a decreased risk of CD and high vegetable consumption with a decreased risk of UC [116]. An additional epidemiological finding is that newly diagnosed paediatric patients with CD were reported to have a lower fruit and vegetable intake compared to healthy subjects [117], though it is not clear whether this constitutes a cause or rather a consequence of IBD.

### 2.2. Intervention Trials with IBD Patients

Unfortunately, there are only a very limited number of human trials available that have focussed directly on IBD with respect to polyphenol intervention (Table 3). Studies in general have incorporated only few subjects, as low as 10 per group, lasting from 4 weeks to 2 years, and included the administration of curcumins, red wine, blueberries, apples, cacao, and pycnogenol, up to approx. 2 g/d. A human study by Chiba et al. [76] with 22 CD subjects showed that a semivegetarian diet, richer in plant foods, and therefore polyphenols, was more successful in maintaining remission over 2 years, compared to an omnivorous diet (94 versus 33%). An earlier trial was conducted with curcumin, a rather apolar polyphenol of limited bioavailability. In their randomized, double blind, placebo controlled multicenter intervention trial, Hanai et al. [75] administered 2 g of curcumins plus medication per day over 6 months to 89 UC patients. A significant improvement in recurrence rate and morbidity parameters associated with UC (clinical activity index and endoscopic index) was found. It cannot be excluded that curcumin, in addition to direct effects, also enhanced the bioavailability of the prescribed medication, due to interactions either at various efflux pumps and/or via altered phase I/phase II metabolism [30].

Koláček et al. [78] investigated the effect of administering pycnogenol, a polyphenolic extract from the maritime pine (*Pinus pinaster*) bark, containing 70% procyanidins, at 2 mg/kg body weight, over 10 weeks to 15 CD patients in remission, and compared the effects to 15 healthy controls. However, controls were not treated, not allowing for an accurate comparison between the groups. Compared to healthy controls, CD patients showed higher levels of Cu/Zn superoxide dismutase (SOD) and increased oxidative damage of proteins. Markers of inflammation such as calprotectin (a protein produced by neutrophils and associated with systemic inflammation) and CRP were negatively associated with total plasma antioxidant activity (TAC). Following intervention, most parameters, including F2-isoprostanes, CRP, and reduced glutathione (GSH), remained rather unchanged when comparing before and after intervention, while lipoperoxide levels and AOPP were significantly reduced, and SOD significantly increased following intervention. Thus, while markers of inflammation remained generally unchanged, markers of oxidative stress were significantly reduced, making this the first study to directly investigate the effects of hydrophilic polyphenols in IBD patients.

Short-term interventions have also been conducted, though with rather more questionable results, in part as inflammation processes are less likely to be altered drastically during short-term trials. However, children with gastroenterological discomfort receiving a novel polyphenol based prebiotic (2 ounces of Preliva (Goodgut INC, USA), rich in Japanese honeysuckle, grape, and pomegranate, among others) within a single dose in a placebo controlled trial experienced significantly less stomach pain and discomfort compared to the placebo group, though no bacteria cultures were measured and no dosing was reported [118]. More such studies, with preferably mid-long-term administration of polyphenols, are much desired.

### 2.3. Studies with Healthy (Non-IBD) Subjects

Other studies have accumulated somewhat more indirect benefits of polyphenols with respect to IBD. In a trial by Clemente-Postigo et al. [79], the effect of red wine (RW), dealcoholised red wine (DRW), and gin consumption on 10 healthy adults was investigated in a randomized cross-over trial over 20 days (272 mL/d wine or 100 mL/d for the gin). Endpoints investigated included serum endotoxin and LPS- (lipopolysaccharide-) binding protein (LBP), in addition to fecal microbiota. No significant differences were detected with regard to serum endotoxin and LBP changes with gin or DRW. However, following RW consumption, numbers of Bifidobacteria and* Prevotella* significantly increased and correlated negatively to LPS levels, emphasizing that soluble phenolic constituents in their natural (i.e., alcoholic) matrix, may improve gut flora in terms of the number of healthy bacteria. However, similar results were obtained in earlier trials, were a (nonalcoholic) cacao-flavonol drink increased Bifidobacteria and Lactobacilli numbers in the gut, reducing CRP (and TG) [77] in the serum, likewise emphasizing potential prebiotic effects of polyphenols, as higher Bifidobacteria numbers have been associated with increased gut barrier properties [119], possibly via their production of SCFA and effects on the gut barrier, reducing, for example, LPS formation [120]. That the effect in the cacao-beverage study was truly attributable to polyphenols is very likely, as a cocoa drink rich in flavan-3-ol was contrasted to a similar cacao drink low in flavon-3-ol.

Another study on obese subjects demonstrated that polyphenols from red wine were able to induce likewise Bifidobacteria and Lactobacilli growth, as well as butyrate producing bacteria [83], reducing LPS producers. However, polyphenols from red wine have also been suggested to hamper inflammatory cytokines in the gut, as found in a subset of healthy volunteers with high cytokine levels (6 out of 34), consuming red wine over 4 weeks (containing ca. 1.76 g/L polyphenols), though the exact amount consumed was not registered. Similarly, in a study with normal healthy (non-IBD) subjects consuming a blueberry beverage rich in polyphenols (375 mg anthocyanins and 128 mg chlorogenic acid per d) for 6 weeks, certain Bifidobacteria counts were more pronounced following the intervention, compared to a placebo drink [80].

Also the consumption of coffee, being rich in polyphenols (in addition to fermentable fibre), has been suggested to enhance the number of health beneficial bacteria. For example, in a study that included administering instant coffee for 3 weeks (3 cups/d) to healthy subjects, the number of Bifidobacteria significantly increased compared to the onset of the intervention [121]. Similar results were obtained for green tea intake [122], enhancing in tendency the proportion of Bifidobacteria, though results for black tea were less clear and did not influence the proportion of Bifidobacteria but rather decreased overall bacteria population [123], showing also high variability of the observed results.

A few reviews have meanwhile also aimed at emphasizing the potential that polyphenols may play regarding the prevention (or as an adjuvant therapy) in IBD [13, 38–40, 124] and even other ailments of the digestive tract, starting with periodontal (gum) applications [125]. In summary however, far too little data exists regarding human trials employing polyphenols in longer intervention studies, also with respect to the kind of polyphenols, dosing, and matrix, to clearly prove that these compounds may prevent or significantly ameliorate the progression of disease, though first trials appear promising.

## 3. Animal Studies and In Vitro Trials

### 3.1. Choice of Model

With respect to animal models, typically mice or rats have been employed due to cost and handling reasons, with colitis being induced by administration of proinflammatory chemicals, mostly dextran sodium sulphate (DSS) or 2,4,6-trinitrobenzenesulfonic acid (TBNA). While animal models can mimic relatively well the inflammation (and oxidative stress) in relation to cytokine activation via molecular targets, the major disparity possibly rests in the microflora, often being different from humans [126]. However, since only about 35% of bacterial genes have been reported to be shared even between human individuals, simulating representatively the microflora is anyhow a difficult task [14]. In addition, chemical induction may not entirely reflect IBDs and all the immunological aspects involved. This may be overcome by the more recent development of genetically modified rodents, which spontaneously develop IBD [127], but those have so far found little application, due to still limited availability.

Regarding in vitro methods, which allow the testing of many dietary factors within a rather short period of time (due to risk of bacterial growth and the need to refresh media), most studies have been conducted with cancer epithelium cells, especially monolayer-forming Caco-2 cells and HT-29 cells, mostly without previous simulation of gastrointestinal digestion, with occasional exceptions [67]. Disregarding digestion processes will bear the risk that changes in the polyphenol profile (see Section 3.2), as well as matrix release, and thus bioaccessibility, are not or only poorly resembling the in vivo situation. However, in these models, inflammation must usually be triggered (due to otherwise very low secretion of, e.g., cytokines), for which certain stimulants are added, often TNF-*α*, IL-1*β*, or LPS or a mixture thereof, even though the activation via LPS has been questioned, due to lack of certain LPS receptors in Caco-2 cells (e.g., TRL4), according to some reports [128].

Thus, the major limitations of these cellular methods are that they (a) typically lack the capability of including the interaction with the colonic microflora, as these are often incompatible with the epithelial cells involved, (b) do not normally include immune cells such as macrophages, which usually do infiltrate inflamed tissues, often aggravating inflammation, (c) only allow rather short-term exposure due to the risk of additional microbiological contamination of the cell models, and (d) do not take into account changes of the polyphenol profile during preceding digestion. However, some more sophisticated models have meanwhile been developed, such as triple-cell culture models encompassing also macrophage-like cells [67], which have been coupled to preceding simulated in vitro digestion, and also models that allow studying the interaction with bacteria and gut cells, such as the microfluidic HUMIX model [129], which however requires complete solubilisation of compounds and works only with small volumes (<100 *μ*L).

### 3.2. Aspects of Digestion and Further Metabolism

As many polyphenols are considerably altered in their structure during digestion, due to either degradation or further active metabolism involving host or microbiota enzymes, it is worth mentioning—at least briefly—major pathways and changes for predominant polyphenols that are consumed. Equally, an understanding of these processes is important as many of the metabolites may have altered bioavailability and/or bioactivity. However, in many in vitro models, such changes are not accounted for, neglecting potential influences of the digestion and/or the microflora on the polyphenol profile, presenting a potential limitation for many investigations.

Following ingestion of the polyphenols, these may or may not be released from the matrix. Possibly, release of the “nonextractable polyphenol fraction,” that is, encompassing especially polyphenols covalently bound to the food matrix, cannot be achieved during gastric and small intestinal digestion but may in part occur in the large intestine [130] following further fermentation of the food matrix. Additional food matrix factors influencing polyphenol bioavailability have been reviewed elsewhere [30]. As many polyphenols are present in the food matrix as glycosides (e.g., flavonoids), these are believed to require (prior to their potential absorption) cleavage by human lactase-phlorizin hydrolase, situated at the brush-border of the intestinal epithelium [131], releasing the free aglycones. Alternatively, cleavage may occur intracellularly by cytosolic beta-glucosidase [132]. In addition, low pH of the stomach may likewise cleave a fraction of the glucosides. Polyphenol esters, such as hydroxycinnamates and diferulates, have also been hypothesized to be cleaved by human enzymes, such as carboxylesterase, present on the brush border or intracellularly within the enterocytes [133].

Regarding bacterial fermentation, which preliminary takes place in the colon, it has been reported that the microbiome is able to result in a multitude of transformations [134], depending on the number and type of bacteria species present, the food matrix, and the type of polyphenols. Mostly, ring fission of, for example, flavonoids [135], demethylation, dehydroxylation, decarboxylation, and deglycosylation and reduction reactions have been reported and have been reviewed previously [136, 137]. Typical end-products may include phenolic acids, or other hydroxylated aromatic compounds [138], which may then be taken up by the colon epithelium. Thus, a number of processes may occur in the upper and lower intestine, which have implications on the polyphenol profile, and therefore on the bioactivity profile, which often are disregarded in simplified in vitro trials.

### 3.3. Antioxidant Aspects—Direct Effects

As many polyphenols can act as radical scavenging compounds and are thus able to act as antioxidants [16], their antioxidant potential has been thoroughly investigated in vitro and in vivo. Strongest antioxidants appear to be compounds with multiple hydroxyl groups, such as flavonoids or tannins [139]. However, bioavailability of many polyphenols may be low. Upon ingestion, polyphenols can be metabolized (deglucosylated, glucuronidated, sulphated, and possibly de-esterified) by human enzymes, with additional changes in the gut by (typically, colonic) bacteria, which may hydrolyse glucosides, glucuronides, sulfates, amides, esters, and lactones and further result in ring-fission, in addition to further reduction, decarboxylation, demethylation, and dehydroxylation, among other reactions [134, 136]. Polyphenols also are typically pumped rapidly out of the cell, often back into the gut lumen, via, for example, multidrug resistant proteins (MRP)1,2,4, P-glycoprotein (P-gp), and breast cancer cell resistant proteins (BCRP), which further reduce their bioavailability. As, following absorption, also urinary excretion may occur rapidly, often following phase II metabolism (sulfation, glucuronidation), this finally results in quite low levels of detectable circulating polyphenols, especially native ones. In addition, as many other systems in the human body can act as radical quenching compounds, including enzymes such as SOD and GPx, other exogenous compounds (vitamins E and C, carotenoids), and many additional endogenous molecules (uric acid, albumin), the overall contribution to direct antioxidant effects therefore appears low [16]. However, polyphenols may have a role either via

- their action as antioxidants prior to absorption, that is, directly in the gut lumen, where their concentration is comparatively high, quenching ROS occurring in the gut lumen, or
- following absorption, via their influence on nuclear receptors and gene expression.

Unfortunately, not much is known about their possibility to quench ROS or reactive nitrogen species (RON) in the gut lumen prior to absorption, as this has never been systematically studied. As it is clear and has been demonstrated that the antioxidant potential of polyphenols prevails, at least in part, during digestion, depending mostly on release kinetics and possible solubility, that is, bioaccessibility, polyphenols can therefore contribute to antioxidant activity [16, 140] in the lumen of the gut. This may be important, as even for the extracellular space (i.e., gut lumen), ROS and RON may be released following inflammatory diseases into the gut, and quenching these species may reduce further aggravation of IBD conditions. However, the potential resulting health benefits in this respect have never been studied and may be more difficult to distinguish from effects following absorption and to extrapolate to the long-term effects in vivo.

### 3.4. Antioxidant Aspects—Effects via Altering Molecular Targets

#### 3.4.1. Animal Trials

In contrast to effects prior to polyphenol uptake (i.e., their activity in the lumen), effects following their absorption and their influence on gene expression via molecular targets (e.g., transcription factors) have been investigated in more detail [141, 142], with mechanistic insights from animal and cellular models.

Table 4 gives an overview on frequently applied animal models. As can be seen, most studies have been finding positive effects based on intervention with various polyphenols and polyphenol rich sources, such as apples, green tea, cacao, pomegranate, and grape seeds, regarding the development of IBD, typically tested by “soft markers,” such as cytokine formation or other inflammation and oxidative stress related aspects, both locally and systemically, in conjunction with histological examinations. Regarding markers of oxidative stress, polyphenols have been shown to modify the formation of MDA [87, 89, 91], hydrogen peroxide [98], protein oxidation [91], and several genes in the mucosa involved in antioxidant defence and detoxification, including, for example, glutathione peroxidase 1 (GPx-1), NAD(P)H dehydrogenase [quinone-1] (NQO-1), peroxiredoxin-6 (PRDX-6), superoxide dismutase 1 (SOD-1), catalase (CAT), and thioredoxinreductase-1 (TXNRD-1) [87, 95], in various rodent models, also confirmed in a study with healthy pigs receiving grapeseed and grape-marc extracts (1% in the diet) for 4 weeks, compared to control pigs. Mechanisms involved appeared to be related to the deactivation of further upstream targets, especially Nrf-2 [95], due to a high antioxidant effect of the extracts; at least this mechanism appears plausible. Often however, Nrf-2 is upregulated following higher doses of individual polyphenols, especially if oxidative stress levels are high. This has been corroborated by several studies, for example, in rats where gut inflammation and oxidative stress were induced by ketoprofen (nonsteroidal anti-inflammatory), receiving catechins (35 mg/kg per day) for 21 d, resulting in increased formation of Nrf-2 downstream targets, that is, glutathione (GSH, reduced form), and also in reduced lactate dehydrogenase (LDH) leakage and 8-hydroxy-guanosine (8-OHdG) [96].

The reduction of oxidative stress and inflammation in the gut has also been reported to have somewhat more systemic effects. In a study by Cazarin et al. [97], administration of* Passiflora edulis* peel rich in fiber and polyphenols for 7 d at 25 g/kg flour reduced serum antioxidant activity (FRAP), GPx, thiobarbituric acid reactive substances such as MDA (TBARS), and glutathione reductase (GR). In another study with mice, green tea polyphenols given for 10 d (no dose specified) enhanced blood levels of GSH [85], and green tea polyphenols or EGCG (epigallocatechin gallate) at 0.25, 0.5, and 1% added to the diet for 10 weeks improved colonic and hepatic GSH in a similar model [58].

Typically, doses of polyphenols or extracts have been ranging between 10 and 20 mg/kg body weight of animals, though lower ones down to 0.5 mg/kg and higher ones up to 100 mg/kg or even 500 mg/kg for certain extracts have been administered (Table 3). Strictly up-scaling these concentrations to humans would result in doses of 700–1400 mg, which is about the daily intake of polyphenols, being high, but achievable, surely with dietary supplements, while doses above would represent supraphysiological amounts. When however taking into account body surface area (BSA), and applying the human equivalent dose (HED), the typical dose applied to animals would translate into approx. 190–380 mg [143], given that the HED (mg/kg) equals animal dose (mg/kg) × (animal Km)/(human Km), with Km being a conversion factor, typically 57 for a human adult and 3 for a mouse (6 for a rat). However, it can be stated that most administered doses are indeed physiologically realistic and are within the daily human intake. Times of administration usually ranged from about 1 to 12 weeks or so, reflecting a considerable lifespan for small rodents.

#### 3.4.2. Cell Culture Studies

The effects observed in animal models are generally confirmed by cell culture studies (Table 5). For example, following digestion of a raspberry extract, the amount of ROS produced due to acrylamide-induced toxicity on Caco-2 cell mitochondria was significantly reduced [144]. More specifically, intracellular ROS generation was lowered, as was mitochondrial membrane potential (MMP) collapse as well as glutathione (GSH) depletion.

In a study applying red wine extract rich in catechin B1 and malvidin-3-glucose on HT-29 cells for 24 h, both COX-2 expression and protein tyrosine nitration, a biomarker of RON, were significantly reduced [69]. In another study, apple peel polyphenols (250 *μ*g/mL for 24 h) reduced lipid peroxidation in Caco-2 cells [111]. At least some antioxidant effects observed in vivo may be ascribed to reduced neutrophil activity, which may produce several types of ROS, as shown by decreased production of ROS in neutrophils, following incubation with the ellagitannin metabolite urolithin B in vitro [145], possibly via inhibiting myeloperoxidase.

It has to be mentioned that some reports (though the minority of published results) did not confirm positive effects of polyphenols on Nrf-2 or downstream targets. In a study with grapeseed and grape-marc extracts rich in polyphenols, employing (TNF-*α* induced) Caco-2 cells exposed for 24 h at 2 mg/mL, no effects on Nrf-2 transactivation or target genes (GPx-2, NOQ1, CP1A1, and UGT1A1) were found [110]. In contrast, administration of a polyphenol-rich plum digesta to a Caco-2/HT-29 (apical) and THP-1 like macrophage (basolateral compartment) model (stimulated with a mixture of LPS, TNF-*α*, and IL-1*β*) for 24 h even reduced transactivation of Nrf-2, possibly indicating reduced oxidative stress levels [67] following polyphenol exposure. These effects could well be concentration dependent, as it has been suggested that certain antioxidants such as retinoic acid at high concentrations may trigger Nrf-2 translocation to the nucleus, while lower, more physiological concentrations, and perhaps employing stimuli not causing excessive oxidative stress responses, had no or even opposite effects [146], being in line with an overall reduced ROS level.

It can thus be speculated that at least in the epithelium (where concentrations of polyphenols may still be reasonably high compared to the deeper cell layers of the intestine) both direct antioxidant effects and more indirect effects, activating the cell's own antioxidant system, may play a role. Again, concentrations at the basolateral side are possibly lower due to the efflux of certain polyphenols by the mentioned transporters back into the lumen. However, it must also be stated that many studies have been applying relatively high doses of polyphenols and extracts to the epithelium, often 25 *μ*M (e.g., resveratrol, i.e., ca. 6 mg/L) or even up to 100 mg/L for other compounds, reachable perhaps via supplements, but not easily with regular food items. It may be argued that, as more long-term effects can normally not be studied with monolayer cell culture models, higher concentrations may somewhat counterbalance for shorter exposure times (not considering additional model limitations such as missing digestion, form of application, i.e., bioaccessibility), though again (as mentioned above) effects of polyphenols may be well concentration dependent.

In conclusion, there is strong evidence from animal trials and in vitro (cellular) experiments that polyphenols, when applied in considerable, but still physiologically relevant doses, do reduce oxidative stress in colonic epithelial cells and tissues stimulated for oxidative stress/inflammation and that the mechanism of oxidative stress is, at least in part related to the transcription factor Nrf-2, influencing further downstream targets.

### 3.5. Anti-Inflammatory Aspects

#### 3.5.1. Animal Trials

**Figure 3:**
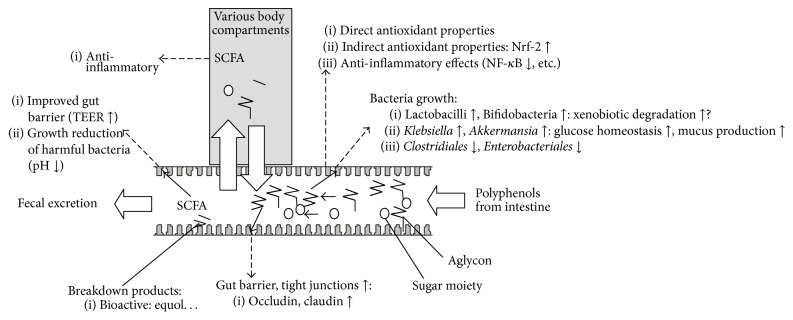
Potential effects of polyphenols on the gut microbiota and possible resulting health effects. SCFA: short-chain fatty acid (butyrate, propionate, and acetate). TEER: trans-epithelial electrical resistance.

While antioxidant effects surely do play a role in the origin and progression of IBD, more attention has been given to inflammatory aspects, possibly as a reduction in inflammation would likely also reduce ROS, and due to the meanwhile reasonably well understood molecular mechanisms underlying IBD, especially the involvement of NF-*κ*B and its further downstream targets (Figure 1). However, also the JAK-STAT (janus kinase and signal transducer and activator of transcription) pathway may be involved, activated by interleukins/interferons, especially in cells of the immune system, resulting, for example, in the activation of apoptotic regulators, such as bcl-XL (b-cell lymphoma extra-large, a transmembrane molecule in the mitochondria, acting as a prosurvival protein) of MYC (encoding for a nuclear phosphoprotein important for cell cycle progression and apoptosis), or alterations of the p21 antitumor progression gene [147]. A limited number of studies have included endpoints related to JAK/STAT. Barnett et al. reported anti-inflammatory activity mediated by multiple molecular pathways, including PPAR-*α* and STAT1, following administration of 0.6% green tea polyphenols for 12 weeks to mice [93]. Lychee polyphenols (5 mg/kg for 2 weeks) significantly reduced STAT3 activation in colon tissue of mice [91], and also adenoma inhibition was observed (Figure 3).

In addition, the MAPK/ERK (mitogen-activated protein kinase/extracellular signal-regulated kinase) pathway, where the phosphorylation of further downstream kinases can result in the activation of apoptosis or altered cell proliferation, is also implicated in many inflammatory related diseases [146] and has been suggested to result in stimulated cytokine production by T-cells [148]. However, few studies have reported MAPK related effects following polyphenol administration. Some polyphenols have been reported to reduce MAPK related signalling pathways in animal trials, including paeonol [149] and genistein [150], while a chalcone derivative [151] enhanced its activity. In a study by Rosillo et al. [86], raspberry polyphenols (10–20 mg/kg for 10 d) reduced activation of p38, c-Jun N-terminal kinase (JNK), and ERK1/2 MAPKs, preventing inhibitory protein I*κ*B-degradation, inhibiting nuclear translocation of p65 (part of NF-*κ*B).

Many studies have meanwhile been performed on animal (typically rodent) models of IBD and intervention with various dietary components, including polyphenol-rich extracts, but also studies employing pure compounds (Table 3). Of these pure compounds, particularly ellagic acid [63, 86], gallic acid [102], naringenin [94], catechin [96], and EGCG [89] have been investigated and associated with anti-inflammatory effects. Thus, it appears that, with respect to anti-inflammatory properties, rather lower molecular weight polyphenols have attracted attention, as opposed to the more complex, that is, higher molecular ones which are regarded as potential prebiotics (see Section 3.5).

However, many extracts and complex food items rich in polyphenols have also been studied, with a focus on green tea [84, 85] and grape constituents [90, 95, 100], though many other food items, including strawberries [87], cranberries [104],* Pepper nigrum* [89], sorghum bran [99], and cacao [105], were also studied. With respect to timing and dosing, polyphenol concentrations ranged from 0.5 to 100 mg/kg body weight for pure compounds, given over 3 days to 12 weeks, constituting high, but still physiologically relevant doses.

Both local effects and systemic effects on inflammation related pathways have been reported. Local effects did include decreased histopathological scores [58, 90, 100], improved length of the colon [94] as a marker of reduced severity of IBD and even reduced weight loss and improved overall survival [89] and inhibition of various cytokine formations such as TNF-*α* [84, 105], IL-6 [102], IL-10 [101], IL-17 [105], IL-1*β* [92, 105], IF-*γ* [101, 102], often linked to reduced expression of NF-*κ*B [86, 91, 95], iNOS [90], and COX-2 [86, 102] (as a prestep to the formation of proinflammatory prostaglandins, e.g., PGE2) and peroxisome proliferator activated receptor- (PPAR-) *α* [93], involved in lipid metabolism, in colonic tissues.

However, reduced cytokine levels in the circulatory system, including TNF-*α* and IL-6 [58], have also been found. Thus, results are in agreement with the theory that polyphenols or their degradation products/metabolites (cleaved aglycones, or glucuronidated and sulfated products) do act on intracellular signaling cascades in the epithelium or in infiltrated immune cells such as neutrophils [86] in the gut, downregulating proinflammatory cytokines, besides the likelihood that the majority of polyphenols is reexcreted into the gut lumen [30]. Whether the systemic measured effects reflect mostly cytokines secreted at the site of the gut, or whether polyphenols also pose considerably anti-inflammatory effects at different sites, is not entirely clear. Since polyphenols have also been reported to reduce inflammation in other chronic inflammation related diseases, such as diabetes [152], both at least appear to be possible. In a study by Skyberg et al. [88], it was also verified whether polyphenols, when given peritoneal, would decrease induced colitis in mice. Contrarily to apple polyphenols given orally, no positive effects however were found, highlighting the importance of oral uptake and direct contact of polyphenols to the epithelial cells, and/or perhaps the prerequisite of forming certain degradation products or metabolites during digestion and/or at the epithelial layer.

The study by Skyberg et al. [88] also highlighted the involvement of T-cells (chemokine receptor CXCR3 expressing TCR*αβ* cells) for apple polyphenol mediated protection, as these cells were indispensable for offering protection against colitis. Previous in vitro studies had already suggested that polyphenols can stimulate natural killer cells and *γδ* T cells (typically found in high abundance in the mucosa), as these cells can upregulate CD69, CD11b, and IL-2R proliferation and induce proinflammatory mRNA transcripts [88]. Thus, also specific immunomodulatory aspects of polyphenols should not be overlooked but have so far received comparably little attention. This is also underpinned by studies showing positive effects of polyphenols on toll-like receptors (TLRs), enhancing the activity of the innate immune system [153]. Also, the excretion of IgA in rats fed with extracts of haskap (honeyberry) and aronia fruits was increased, likewise demonstrating immune-stimulating effects [154].

#### 3.5.2. Cell Culture Investigations

Though cell culture studies suffer various drawbacks, such as allowing studying inflammatory processes only during relatively short-term periods, and often lack the complexity of the in vivo epithelium, that is, the interactions between the various cell types involved in inflammation of the intestine, it is possible to examine many factors in rather short time periods, yielding mechanistic insights into the relation of dietary compounds and inflammatory processes, with or without preceding digestion.

Employing a triple culture model (with Caco-2/HT-29 MTX cells in the apical and THP-1 like macrophages in the basolateral compartment) coupled to preceding simulated gastrointestinal digestion, Kaulmann et al. [67] reported that digested plum extracts (ca. 1.2 g/L wet weight) were able to reduce IL-8 production by Caco-2/HT-29 cells, and several kale extracts reduced IL-6 secretion in THP-1 cells, which was related to reduced NF-*κ*B expression. However, both extracts rich and poor in polyphenols (and in carotenoids) did exert positive effects, suggesting that other compounds at least contributed to the positive effects, such as vitamin C. Romier et al. [107] investigated a variety of polyphenols and extracts with respect to inflammatory endpoints when exposed to Caco-2 cells, finding somewhat ambivalent results. While chrysin and ellagic acid (50 *μ*M) reduced NF-*κ*B expression, resveratrol and genistein increased it. Chrysin, ellagic acid, genistein, and epigallocatechin gallate reduced IL-8 secretion, while again resveratrol promoted it, pointing out that some polyphenols may show arbitrary effects when ingested at high concentrations. However, polyphenols did, as in most studies, not undergo simulated digestion, which on the other hand may not have had drastic effects on the compounds investigated, as these were mostly water soluble (not requiring solubilisation in form of micelles, except perhaps resveratrol) and were administered as aglycones.

Another limitation is that colonic fermentation is usually not coupled to in vitro trials, though possibly strongly affecting polyphenol profile. The difficulty rests again in the noncompatibility of the epithelial cells employed and the bacteria, plus the difficulties to maintain strict anaerobic conditions. Also, very few studies have included colonic metabolites. Miene et al. [108] investigated the effect of quercetin and chlorogenic acid/caffeic acid metabolites (3,4-dihydroxyphenylacetic acid (ES) and 3-(3,4-dihydroxyphenyl)-propionic acid (PS), resp.) on colonic LT97 cells, finding reduced COX-2 expression. A further difficulty rests in the fact that many of the colonic metabolites are not commercially available and therefore remain understudied.

Most cell culture studies have been conducted with pure polyphenols, including especially curcumin, resveratrol [114], genistein, chrysin, and EGCG [107], cyanidin-3-glucoside [112], and catechin, theaflavin, malvidin, cyanidin, and apigenin [96], though extracts, especially red wine [69, 109], apple (peel) [111], blueberry [113], and grape [110], have also been studied. Concentrations of individual compounds, as stated also above, have ranged from ca. 25 to 100 *μ*M, and of extracts up to 600 *μ*g/mL, which is considered high but physiologically reachable in the gut. A drawback of most studies, again, is the missing preceding digestion, which would limit especially the bioavailability of the more apolar polyphenols, namely, resveratrol and curcumin, due to missing emulsification, that is, solubilisation in mixed micelles. Most models have included Caco-2 cells or HT-29 cells, which may underestimate the strength of in vivo responses, as immune cells have mostly not been employed. The majority of these trials have demonstrated that polyphenols or polyphenol-rich extracts were able to reduce proinflammatory cytokines, including typically IL-8 [67, 69, 107, 110, 112], but also PGE-2, TNF-*α*, and IL-1*β*, often both at mRNA expression level and at protein level, and that this was related to reduced NF-*κ*B expression (Table 5).

Other studies have focussed on downstream targets of COX-2. In a study by Serra et al. [112], cyanidin-3-glucose administered for 24 h at 25 *μ*M reduced PGE-2 expression in HT-29 cells, possibly as a consequence of influencing COX-2 expression, which was also detected. Apple peel polyphenols (250 *μ*g/mL) for 24 h also reduced COX-2 activity [111] in Caco-2 cells.

The fact that also intracellular adhesion molecule- (ICAM-) 1 was significantly downregulated by, for example, red wine polyphenols [109], important for leukocyte endothelial transmigration, monocyte chemoattractant protein 1 (MCP-1), and chemokine (C-X-C motif) ligand 1 (CXCL1), also having neutrophil chemoattractant activity, vascular cell adhesion protein-1 (VCAM-1, promoting adhesion of other immune cells), and platelet endothelial cell adhesion molecule 1 (PECAM-1, equal to CD31, playing a role in neutrophil removal), in further studies (Table 4), also suggests, as do animal studies, that modulation of the immune system is also a potential important function of polyphenols. Furthermore, apoptosis may also be influenced, as shown with soy legume extracts on both T84 colon cells and macrophages at over 30 *μ*M [106], where inflammation-related apoptosis in the epithelial cells (induced by peroxynitrites) was significantly reduced, while macrophage viability was compromised.

In conclusion, several cellular trials are in line with animal study findings that both polyphenols and polyphenol-rich products are able to reduce the concentration of proinflammatory cytokines, acting via reduced NF-*κ*B expression and translocation, though additional functions such as modulation of the immune system, reducing, for example, leukocyte transmigration, neutrophil attraction, and finally altered apoptosis, may also play a role.

### 3.6. Gut Microflora

The importance of the gut microbiota has recently been highlighted in a study by Schaubeck et al. [155], where disease associated microbiota was transplanted in a mouse model, causing CD in the transfected mice, clearly demonstrating gut microflora as a causative agent of IBD.

Several studies including cellular, animal, and human trials have indicated that several polyphenols from fruits or vegetables may increase the number of potential health beneficial bacteria in the gut [156–158], thus acting as prebiotics. This in turn is expected to have positive intestinal effects, including, for example, the formation of SCFA, to enhance gut barrier properties, and fostering the growth of potentially less harmful bacteria. Earlier trials in this respect have shown, for instance, that, in Caco-2 cells, propionate, acetate, and especially butyrate (2 mM) increased the transepithelial electrical resistance (TEER) by almost 300% [159] after 72 h of incubation with these SCFAs. As however also DMSO showed similar effects, it was reasoned that further cell differentiation played a major role in reducing permeability. On the other hand, germ-free mice do not develop IBD, and humans treated with antibiotics appear to result in at least temporary remission of IBD [15], emphasizing that the microbiota could act as a double edged sword.

Nevertheless, the question to what extent and via which mechanisms polyphenols may act as prebiotics is only poorly comprehended, though the evidence that polyphenols contribute to the number of health beneficial bacteria such as* Lactobacilli* and Bifidobacteria is increasing [158, 160], while suggesting a likewise reduction in orders including potential pathogenic bacteria such as* Clostridiales* and* Enterobacteriales*, and even LPS or toxin producing* E. coli* strains, such as O157:H7 [161]. It has also been suggested, based on in vitro studies, that the effects are rather related to the aglycones than the glycosides, at least for flavonoids [162], perhaps also emphasizing the role that bacteria could play in deglycosylation of the native glycosides. Altering the gut flora can have percussions on the further degradation of carbohydrates, including potential probiotics. In an in vitro study by Xue et al. [163], the plant polyphenols quercetin, catechin, and puerarin, when added to the cellular media, downregulated the ratio of Firmicutes : Bacteroidetes, which altered the degradation of fructooligosaccharides (FOS). Similarly, in a study with anthocyanin rich strawberry extract [164], the formation of FOS breakdown products and acidification achieved in the gut was increased in rat cecal digesta and urine, also emphasizing the potential positive effect of additional polyphenols and their implication in energy metabolism. In addition to their influence on carbohydrate metabolism, polyphenols have also been reported to influence lipid and provitamin metabolism, via their alteration of gut microbiota, and therefore could also change human homeostasis [165, 166].

Several in vitro studies have pointed out that polyphenol-rich extracts, such as from pomegranate, can enhance the growth of* Bifidobacterium* and* Lactobacillus* [167]. Similar effects in a batch-culture fermentation in vitro model were observed with coffee with high levels of chlorogenic acid [168]. Studies in this domain have been impeded by the fact that many bacteria cannot easily be grown ex vivo, though models simulating also colonic digestion under realistic, that is, low oxygen, environmental conditions, such as the SHIME, have further deepened the understanding of the interrelation between the diet and the microbiota [169], also highlighting that polyphenols can provoke a shift of the microbiota. This included, for example, the growth of* Klebsiella* and* Akkermansia* spp., with the latter growing especially in mucus-rich environment, producing SCFA. Particularly proanthocyanidins, oligomeric flavonoids, rich, for example, in barks of trees but also in grapes, have been speculated to foster the growth of* Akkermansia* [160], as it increased mucus layer thickness, improved glucose homeostasis, and alleviated metabolic endotoxemia. An additional effect may rest in the influence* Akkermansia* appears to exert on branched chain amino acids (BCAA). A low plasma level of BCAA has been associated with reduced insulin secretion and lower weight in obese subjects [170]; however, they may also promote a more systemic proinflammatory response [171].

In addition to causing a shift toward an altered microbiota composition, it appears that polyphenols also induce a transformation in bacterial genes toward xenobiotic degradation, as found in a study on rats receiving blueberry powder for 6 weeks, whereas genes related to BCAA degradation, known to reduce gut atrophy [172], and those associated with higher invasiveness were enhanced and reduced, respectively [161], thus perhaps fostering the deactivation of other harmful xenobiotic compounds and possibly improving gut barrier function.

A few in vivo studies, including animals and humans (see Sections 2 and 3), have confirmed the positive effects that polyphenol-rich extracts may exert toward fostering healthy microflora. In pigs, proanthocyanidins in grapeseed extract (1%) consumed over 6 d enhanced however* Lachnospiraceae*,* Clostridiales*,* Lactobacillus,* and* Ruminococcaceae* [173], also showing that effects are surely model and polyphenol dependent. C57BL/6J mice kept on a high fat/high sucrose diet receiving either water or cranberry extract (200mg/kg) for 8 weeks showed an enhanced population of* Akkermansia*, in addition to reducing intestinal oxidative stress and inflammation [104]. Similar effects (enhanced Bacteroidetes/Firmicutes ratio, often associated with less obese subjects; reduced* Bacillus*; increased* Akkermansia muciniphila*) were also observed when giving resveratrol (15 mg/kg bw.) or quercetin (30 mg/kg bw.) to rats for 6 weeks [174], highlighting the effects of pure polyphenols not associated with dietary fiber. Similarly, administration of a carbohydrate-free plum or peach extract (430 and 1,270 mg GAE (gallic acid equivalents)/mL, resp.) to obese sugar rats for 11 weeks resulted in increased abundance of* Lactobacillus* and members of* Ruminococcaceae*, especially in the more polyphenol-rich plum extract.

In conclusion, several in vitro, animal, and even human trials have suggested that polyphenols, in physiological doses, that is, achievable via a diet rich in fruits and vegetables, or with supplements, over the time-course of a few weeks, are able to shift the microbiota toward a more presumably healthy one, with an enhanced population of Bacteroidetes, producing SCFA, and perhaps being capable of a faster degradation of certain xenobiotic compounds.

### 3.7. Epithelium Protection and Other Aspects

In addition to strengthening intestinal barrier properties via fostering the growth of health beneficial bacteria (see Section 3.6), polyphenols have further been proposed to influence directly the permeability of the mucosa, via acting on the tight junctions, composed especially of occludin and claudin proteins. In a study by Carrasco-Pozo et al. [175], several polyphenols, that is, quercetin, epigallocatechin gallate, and resveratrol (though not rutin), protected tight junction integrity in a Caco-2 based model, via inhibiting the redistribution of the zonula occludens- (ZO-) 1 protein induced by indomethacin and preventing the decreased expression of ZO-1 and occludin caused by indomethacin, possibly related to the polyphenol capacity to protect the mitochondria and reduce ATP depletion. Similarly, in a study employing Ussing chambers and T84 monolayers, polyphenols (ferulic and isoferulic acid, but not caffeic or p-coumaric acid) reversed the negative effect of sodium caprate on tight junction functionality [176], as measured by TEER. This effect was ascribed to the increased expression of tight junction components of ZO-1 and claudin-4 transcription and reduced occludin expression. In a similar study employing T84 cell monolayers, the negative effects of sodium caprate on tight junction associated genes were counterbalanced by several apple polyphenols and their presumed intestinal digestion products, including caffeate, quinic acid, and methyl-p-coumarate [177]. In a study with Caco-2 cells, quercetin (and its metabolite 3,4-dihydroxybenzoic acid) enhanced epithelial resistance to 157 (and 119%, resp.), of control TEER values, which was related to an increased expression rate of claudin-4 [178]. Positive effects on cellular barriers in vitro were also found following cayenne pepper and paprika exposure (reviewed by [179]), though these may have contained considerable amounts of other bioactive compounds, such as vitamin C and carotenoids.

However, also negative effects on tight junctions have been reported, for example, when giving ochratoxin together with polyphenols from (dealcoholised) red wine, via enhanced intracellular redistribution of claudin-4 [180], perhaps as a result of increased uptake and/or reduced excretion and/or metabolism of ochratoxin in the presence of polyphenols.

These, at least in part, positive findings of polyphenols are corroborated by animal studies. In a study by Yang et al. [100], grape seed polyphenols (1% dry weight added to diet given for 16 weeks) improved claudin-2 protein and increased barrier forming claudin-1 protein expression in IL-10 deficient mice, occasionally employed as a model of IBD. Reduced colonic permeability as measured in everted colons by a fluorescein dye was found in colitis induced mice receiving 0.3% naringenin for 9 d in the diet [94]. In a study on rats, curcumin was able to protect the nephron from the negative effects of cisplatin, an anticancer drug, improving stability of tight junctions via enhancing the expression of adherens junction proteins occludin, claudin-2, and E-cadherin [181]. Likewise, in a study with rats investigating neuroprotective effects, subjected to occlusion of the cerebral artery, receiving green tea polyphenols (400 mg/kg and day for 30 d), decreased mRNA/protein expressions of claudin-5, occludin, and ZO-1 in microvessels of ischemic tissue were prevented [182]. Thus, taken together, the results suggest that polyphenols can improve gene expression related to the production of proteins required for tight junction integrity, including possibly claudin-5, occludin, and ZO-1, and that these effects may not be limited to the gut epithelium only.

An additional effect of barrier protection may also rest in increased mucus production or a more stable mucus layer. As also the mucus may have an important barrier and protection function, limiting direct contact of potential proinflammatory stimuli with the epithelial cells, mucus production was likewise investigated in several studies. Rosillo et al. [86] scrutinized the effect of ellagic acid (10–20 mg/kg) in a rat model with TBNS induced colitis. Among others, enhanced mucus production by goblet cells in the colon mucosa was found. Likewise, B proanthocyanidin-rich extracts were suggested to increase mucus secretion, in turn creating a suitable environment for* Akkermansia*, which may further foster SCFA production [80]. Dietary polyphenols have also been stated to be able to cross-link mucin, enhancing the viscoelastic modulus of the mucus layer [183], stabilizing the mucus layer in the intestine [184]. Nevertheless, more studies in this domain are required.

## 4. Potential Arbitrary Effects of Polyphenols or Absence of Effects

With respect to at least IBD, despite the fact that there is much evidence accumulating emphasizing the potential health benefits of polyphenols, it should not be overlooked that polyphenols may also have arbitrary effects on the gut epithelium and the host in general. In addition, there is still a general paucity with respect to robust human trials (i.e., placebo controlled randomized intervention trials) clearly demonstrating positive health effects of polyphenols regarding IBD development and/or progression. In addition, several studies have also cast some doubts that polyphenols, at least alone, are truly the (sole) bioactive agents. For example, in a study with plums and cabbages with contrasting polyphenol and carotenoid profile, concentrations of these phytochemicals were not related to higher anti-inflammatory bioactivity in an in vitro model of digestion and inflammation, suggesting that other bioactive compounds, including dietary fiber or vitamin C, may also contribute to the observed effects regarding, for example, cytokine (IL-8, IL-6) reduction, NF-*κ*B, and Nrf-2 translocation [67]. Finally, it should also be considered that many trials have been conducted in vitro with native components, without preceding digestion and colonic fermentation, which are not likely to be present in the colon under in vivo conditions, and not at the rather high concentrations often employed. Furthermore, polyphenols may, in response to dose and nature, enact negative effects via the following pathways:

- They could act as prooxidants, especially when given isolated and in high doses.
- They may perturb absorption of other bioactive compounds, such as drugs or other phytochemicals.
- They may interact and/or saturate pathways related to phase I/II metabolism, likewise increasing the concentration of otherwise more highly metabolised bioactive compounds.
- They may have other negative effects following bacterial metabolism.

With respect to acting as prooxidants, it has been highlighted that especially compounds with several free hydroxyl groups, such as flavonoids, can, in the presence of free metal ions such as copper or iron, released, for example, when tissue is damaged (expected in subjects with IBD), act as prooxidants, via the Fenton reaction (producing peroxides), as reviewed previously [16, 185]. For some compounds, such as for quercetin, adverse effects at higher concentrations have indeed been shown, especially if reduced glutathione is already low and ROS level already high, as the oxidized quercetin-quinone product will then react with other thiol groups (e.g., from enzymes) [186]. Rat feeding experiments with quercetin have corroborated these results, showing decreased hepatic glutathione concentration and glutathione reductase when receiving 20 mg quercetin/day for up to 6 weeks [187]. Thus, type and dosing of polyphenols should be carefully considered for subjects already showing oxidative stress, such as for smokers, but maybe also for IBD patients. It should also not be overlooked that the administration of other antioxidants, such as beta-carotene, though being much more apolar, has been suggested to cause detrimental health effects in human meta-analyses [188].

As stated, it has to be considered that polyphenols may also block certain efflux transporters in the gut epithelium [30] and may increase the uptake of toxicological relevant compounds, therefore constituting a double edged sword. For example, in a study with Caco-2 cells, dealcoholised red wine aggravated the permeability of the monolayer when ochratoxin was also given [180], even though a recent study on rats did not detect significantly altered toxicokinetics in rats receiving both ochratoxin and quercetin [189]. The potential interactions between secondary plant compounds and other xenobiotics have been highlighted in an earlier review [190].

Similarly, phenol rich matrices such as grapefruit juice or green tea extracts have been hypothesized, due to their high content of polyphenols (naringenin and catechins, resp.), to reduce certain phase II metabolising enzymes (e.g., cytochrome P-450 (CYP) 3A4 isoenzyme), increasing the concentrations of unmetabolized drugs such as statins or antihistamines [191, 192], though they may in addition also reduce efflux-transporter activity such as P-gp, likewise increasing their apparent (absorbed) dose. The same mechanism has been suggested to contribute to high bioavailability of curcuminoids when piperidine (present in, e.g., black pepper) is simultaneously administered [193].

Finally, bacterial metabolites of polyphenols have been reported to also have potential negative effects. Following quercetin and rutin metabolism, the produced 3,4-dihydrophenylacetic acid (DOPAC), which was reported to show anticancer and anti-inflammatory properties, may also inhibit mitochondrial respiration, though this has been rather shown for brain mitochondria [156], nevertheless also suggesting that the therapeutic window may be limited, and that higher concentrations of certain compounds or metabolites could cause enhanced cellular damage. Other compounds, including resveratrol and genistein, have, when administered to cell models at high but physiological concentrations (50 *μ*M), enhanced NF-*κ*B expression, suggesting proinflammatory behaviour.

## 5. Conclusions

Many animal and in vitro (cellular) experiments have shown and emphasized positive effects of polyphenol-rich plants, their extracts, and also individual compounds, on ameliorating the severity and progression of IBD. It appears that polyphenols may not be the sole constituents with health beneficial properties in extracts or more complex matrices, but that other compounds, such as dietary fiber, or vitamin C, may also have certain effects. Nevertheless, it appears likely that, by themselves, polyphenols can exert positive effects, reducing oxidative stress caused by or aggravated by infiltrating neutrophils and macrophages, and are able to locally reduce inflammation, most likely via acting on molecular targets such as NF-*κ*B (related to inflammation) and Nrf-2 (related to oxidative stress), with the latter mechanism requiring cellular uptake into the epithelium. In addition, several studies have suggested prebiotic like effects, fostering the growth of healthy microflora (e.g., Bacteroidetes), which may have anti-inflammatory effects, for example, via SCFA production, or aiding in stabilizing barrier properties, which may also occur via direct effects on claudins and occludins or, alternatively, on the mucus layer.

Nevertheless, administering high doses of polyphenols may also pose a certain risk to subjects already suffering from oxidative stress and inflammation, as polyphenols could also act as prooxidants, perhaps especially when administered in high individual doses. Finally, due to their efflux-altering properties and effects on various CYP metabolising enzymes, interactions with drugs and other xenobiotics should be carefully considered. In addition to more sophisticated cellular models and enhanced commercial availability of colonic metabolites, more human trials are needed to confirm that polyphenols could in fact constitute a preventive strategy and/or supplementary treatment for subjects suffering from IBD and whether individual polyphenols or rather complex mixtures such as extracts are more potent and promising in order to ameliorate this ailment.

## Figures and Tables

**Table 1:** Overview of major risk factors and suggested mechanisms involved in the development of IBD.

Risk factor	Influence: positive (+), negative (−)	Mechanism	Reference
Genetic predisposition	+/−	Genes involved in inflammation and oxidative stress responses and in immune function (histocompatibility complex)	[19, 33]

Smoking	−	Altered blood flow, enhanced cytokine formation, immunomodulatory, influencing mucus production	[18]

Air pollution (NOx, SO_2_…)	−	Unclear: proinflammatory response to air particles?	[18]

Enhanced hygiene	−	Unclear: reduced exposure early in life to microorganisms. Reduced IBD prevalence found for growing up on farms, living in crowded homes, consuming unpasteurized milk	[18]

Microbiota	+/−	Immunomodulatory properties, production of anti-inflammatory compounds. Some bacteria strains associated with negative effects (e.g., Clostridia), others with positive effects, such as Bifidobacteria, possibly due to enhanced gut barrier properties, production of SCFA	[34]

Diet, probiotics	+	Immunomodulatory properties, production of anti-inflammatory compounds, see microbiota	[28]

Diet, prebiotics (fiber)	+	Favouring healthy microbiota (Bifidobacteria…), production of anti-inflammatory SCFA, lowering of pH	[28]

Diet, vitamin D	+	Immunomodulatory, protection of barrier	[35]

Diet, dietary fiber	+	Production of anti-inflammatory SCFA, increasing fecal bulk and lowering concentration of compounds with adverse effects	[17, 26]

Diet, vitamins C, E	+/−	Unclear: antioxidants may reduce ROS parameters, normalize abnormally low tissue levels in subjects with IBD	[36]

Diet, PUFAs (*ω*-3)	+	Anti-inflammatory effects	[37]

Diet, polyphenols	+	Antioxidant effects (reducing ROS), anti-inflammatory properties, altering genetic expression via NF-*κ*B, Nrf-2, improving barrier properties, immunomodulatory	[13, 38–40]

PUFAs: polyunsaturated fatty acids, ROS: reactive oxygen species, and SCFA: short-chain fatty acids.

**Table 2:** Selected food items rich in various polyphenol classes, including potential health beneficial effects.

Food item	Edible part	Concentration (mg/100 g)^**∗**^	Major polyphenol (classes)	Reference regarding content	Suggested health effects, selection^§^	Reference regarding health effect
Apples	Peel	50–120^$^	Phlorizin, phenolic acids: chlorogenic acid, quercetin	[41]	Blood glucose lowering, anti-inflammatory, lowering colonic inflammation	[42–44]
	Flesh	0.2–0.9				
	Total	ca. 5–50				

Blackberries	Whole	130–405	Anthocyanins, flavanols: EC, phenolic acid: ellagic acid	Phenol explorer^1^	Anti-inflammatory, anti-IBD	[45, 46]

Blueberries, highbush	Whole	160–480	Anthocyanins, flavonols: quercetin, phenolic acids: chlorogenic acid	Phenol explorer	Anti-inflammatory, anti-IBD	[47]

Cacao	Bean, powder	300–1100	Flavanols: EC	Phenol explorer	Anti-inflammatory, against heart failure	[48, 49]

Chestnut, raw	Whole nut	547–1960	Hydroxybenzoic acids: gallic acid, ellagic acid, tannins	Phenol explorer	Anti-inflammatory	[50]

Chocolate	Dark	150–425	Flavanols: epicatechin, hydroxycinnamic acid: ferulic acid	Phenol explorer	Reducing CVD, anti-inflammatory	[51]

Cloves	Seasoning, dried	1200–17500	Hydroxyhenylpropenes: eugenol, acetyl eugenol	Phenol explorer	Anti-inflammatory, antiulcer	[52]

Coffee	Beverage, filtered	90	Phenolic acids: chlorogenic acid	Phenol explorer	Improved blood lipids, improved glucose handling, anti-inflammatory but increases IBD symptoms	[53, 54]

Curcuma	Spice, whole	200^+^	Curcuminoids, flavonoids, phenolic acids	[55]	Anti-IBD, anticancerogenic, anti-inflammatory	[56]

Grapefruit	Flesh	15–115	Flavonoids, phenolic acids	Phenol explorer	Anti-inflammatory	[57]

Green tea	Drinkable extract	29–103	Flavanols: EC, EGCG	Phenol explorer	Anti-inflammatory, anticolitic	[58]

Olive oil, extra virgin	Whole oil	4–200	Tyrosols, lignans: pinoresinol; phenolic acids, hydrolysable tannins	Phenol explorer	Anti-inflammatory, reducing CVD	[59]

Paprika, green	Whole fruit	0.3–10	Flavonoids: luteolin glucosides; hydroxycinnamic acids	Phenol explorer	Anti-inflammatory	[60]

Peppermint	Seasoning, dried	450–26000	Flavonoids: eriocitrin; hydroxycinnamic acids: rosmarinic acid	Phenol explorer	Anti-inflammatory	[61]

Pomegranate	Juice	240	Punicalagin (an ellagitannin)	[62]	Anti-inflammatory, anti-IBD	[63]

Potato	Peel Flesh	180–5000^#^ 1–1000^#^	Phenolic acids: chlorogenic acid	[64]	Glycoalkaloids may increase IBD, anthocyanins anti-inflammatory effects in colored potatoes	[65, 66]
	Total	10–50		Phenol explorer		

Plum, dark	Total	130–240	Phenolic acids: chlorogenic acid; procyanidins, anthocyanins	Phenol explorer	Anti-inflammatory, antioxidant	[67, 68]

Red wine	Final product	25–300^**∗**^	Phenolic acids, anthocyanins, tannins, stilbenes (resveratrol)	Phenol explorer	Improved blood lipids, anti-inflammatory, anti-IBD	[69, 70]

Soy	Flour	140–900	Isoflavonoids: daidzein, glycitein, genistein, & glucosides	Phenol explorer	Improved blood lipids, antiapoptotic effects, anti-inflammatory, anti-IBD	[71, 72]

Spinach	Leaf	30–290	Flavonols	Phenol explorer	Anti-inflammatory, anti-IBD	[73]

Wheat	Whole grain	85–220	Phenolic acids: hydroxybenzoic acids, hydroxycinnamic acids	Phenol explorer	Unclear, enhancing celiac disease, controversial effects	[74]

^**∗**^In juices and wine: mg/100 mL; ^1^ http://phenol-explorer.eu/; ^#^calculated from dry weight assuming 80% water content. Note that content in purple potatoes is ca. 5 times higher in polyphenol content than other varieties; ^$^concentration in mg/cm^2^, CVD = cardiovascular diseases, EC = epicatechin, and EPGC = epigallocatechin; ^+^total polyphenols with Folin-Ciocalteu; ^§^effect refers to observation with whole food or respective extracts but is attributed—at least in part—to the respective polyphenols.

**Table 3:** Human intervention trials suggesting health benefits of polyphenol intake with respect to IBD—an overview.

Aspects studied	Dosing and time	Effects found	Mechanism proposed and critics	Reference
Effect of curcumin on CD patients in a RCT study	89 patients with UC, 45 given 2 g curcumin/d for 6 months	Curcumin improved both clinical activity index & endoscopic index	Curcumin suppressed morbidity associated with UC	[75]

Effect of different dietary interventions, prospective trial	22 CD subjects receiving semivegetarian versus omnivorous diet for 2 y	Semivegetarian diet more successful in maintaining remission over 2 y versus omnivorous diet (94 versus 33%)	Effects of fiber & polyphenols?	[76]

Effect of cacao drink on gut bacteria	22 healthy adults receiving 494 mg or (*n* = 23) cocoa flavanols/d for 4 weeks	Significant reductions in plasma TG & CRP in group receiving high flavonol drink	Flavonol rich drink sign. increased Bifidobacteria & Lactobacilli populations, sign. decreasing Clostridia counts	[77]

Effect of pycnogenol in subjects with CD	15 children with CD receiving 2 mg/kg bw. for 12 weeks. 15 control children with no intervention	Compared to baseline, improved lipoperoxides, improved SOD, reduced AOPP	Oxidative stress related markers improved following PP consumption	[78]

Effect of red wine consumption on plasma LPS & gut bacteria	10 volunteers, 20 d, 272 mL of red wine (RW) with/without alcohol (DRW), or 100 mL gin	No significant differences in the change in LPS or LBP conc. between chronic RW, DRW, & gin consumption	*Bifidobacterium* & *Prevotella* amounts were significantly increased by RW & correlated negatively with LPS concentrations	[79]

Effect of blueberry beverage (bb) on healthy subjects	20 subjects, either consuming bb (375 mg ACNs & 128 mg CA) for 6 weeks versus placebo	Bb enhanced Bifidobacteria counts compared to placebo	Prebiotic effect of polyphenols	[80]

Effect of apple products on inflammation & gut microbiota	5 × 4 weeks crossover, whole apples (550 g/d), apple pomace (22 g/d), clear & cloudy apple juices (500 mL/d), none, *n* = 23 healthy adults, 75–240 mg PP/d	No effect on HDL-cholesterol, TAG, weight, waist-to-hip ratio, blood pressure, CRP, gut microbiota, insulin, IGF1, lower serum LDL for whole apples & pomace	Too high variation of bacterial changes such as Bifidobacteria, already health subjects, too short intervention period?	[81]

Effect of red wine on fecal markers of inflammation	34 healthy subjects drinking RW (1.76 g/L PP) for 4 weeks	In a subgroup of 6 subjects, TNF-*α*, IL-6, & IFN-*γ* in feces were sign. reduced	Reduced inflammation via NF-*κ*B?	[82]

Effect of red wine PP on gut bacteria of obese subjects	10 obese & 10 normal subjects receiving 272 mL RW over 30 d	PP sign. increased fecal Bifidobacteria & Lactobacillus & butyrate producers (*Faecalibacterium prausnitzii* & *Roseburia*) at expense of undesired bacteria, for example, LPS producers (*E. coli* & *Enterobacter cloacae*)	Intestinal barrier protection & SCFA production	[83]

ACNs: anthocyanins; AMPK: adenosine monophosphate kinase; AOPP: advanced oxidation protein end-products; Bcl-xl: B-cell lymphoma-extra large; bcrp: breast cancer cell resistance protein; bw: body weight; CA: chlorogenic acid; CAT: catalase; ccl2: chemokine (C-C motif) ligand 2; CINC1: cytokine-induced neutrophil chemoattractant-1; CYP1A1: cytochrome P450, family 1, member 1A1; CD: Crohn's disease; COX-2: cyclooxygenase 2; CRP: c-reactive protein; CXCL1: chemokine (C-X-C motif) ligand, neutrophil activating; DAI: disease activity index; DSS: dextran sodium sulphate; DRW: dealcoholized wine; EA: ellagic acid; EGCG: epigallocatechin gallate; EP: evening primrose (*Oenothera paradoxa*) pomace; GAE: gallic acid equivalents; GM-CSF: granulocyte macrophage colony stimulating factor; FRAP: ferric reducing antioxidant power assay; GR: glutathione reductase; Gred: reduced glutathione; GrTP: green tea extract; GPx: glutathione peroxidase; GSH: glutathione (reduced); GSP: grape seed polyphenols; GSTT2: glutathione-S-transferase theta 2; ICAM: Intercellular adhesion molecule 1; IFN: interferon; IL: interleukin; iNOS: inducible nitric oxide synthase; LBP: LPS binding protein; LPS: lipopolysaccharides; Mcp-1: monocyte chemoattractant protein-1; Mdr1a (−/−): multidrug resistance targeted mutation; MDA: malondialdehyde; MPO: myeloperoxidase; MIP2: macrophage inflammatory protein 2; MRP: multidrug resistance protein; OHdG: 8-hydroxy-2′-deoxyguanosine; NF-*κ*B: nuclear factor kappa B; Nrf-2: nuclear factor (erythroid-derived 2)-like 2; NQO1: NAD(P)H dehydrogenase [quinone-1] 1; PCAM-1: platelet endothelial cell adhesion molecule; PGE2: prostaglandin-E2; P-gp: P-glycoprotein; PP: polyphenols; PRDX-6: peroxiredoxin-6; PPAR: peroxisome proliferator-activated receptor; RCT: randomized control trial; RW: red wine; SAA: serum amyloid *α*; SAPK: stress activated protein kinase; SCFAs: short chain fatty acids; SOD: superoxide dismutase; STAT1: signal transducer and activator of transcription 1; TAC: total antioxidant capacity; TBNS: 2,4,6-trinitrobenzenesulfonic acid; TBARS: thiobarbituric acid reactive substances; TG: triglycerides; TNF-*α*: tumor necrosis factor alpha; TXNRD-1: thioredoxin reductase-1; UGT1A1: UDP-glucuronosyltransferase family 1 member A1; UTR: untranslated; VCAM-1: vascular cell adhesion protein 1; WB: Western blot.

**Table 4:** Animal trials suggesting positive health benefits of polyphenols with respect to IBD—an overview.

Aspects studied	Dosing and time	Effects found	Mechanism proposed and critics	Reference
Effect of green tea PP on DSS induced colitis in IL-2 deficient mice	Water with 5 g/L green tea PP for 6 weeks	Reduced serum amyloid A, increased weight gain & hematocrit, reduced IFN-*γ*, TNF-*α* in cultured cells from colon	Anti-inflammatory effects of green tea PP	[84]

Effect of green tea PP & other antioxidants on DSS induced colitis in mice	10 d, no dose specified	Lengthening of colon, enhanced blood level of reduced GSH, improved serum amyloid A, TNF-*α*, improved cytoskeleton	Improved antioxidant status	[85]

Effect of ellagic acid (EA) on rats with TBNS induced colitis	10-11 rats per group receiving 10–20 mg/kg EA for 10 d	EA decreased neutrophil infiltration & COX-2 & iNOS. Reduced activation of p38, JNK & ERK1/2 MAPKs, preventing inhibitory protein I*κ*B-degradation, inhibiting nuclear translocation of p65	EA diminished severity & extension of intestinal injuries. EA also increased mucus production in goblet cells in colon mucosa	[86]

Effect of strawberry PP on rats with induced gastric lesions	40 mg/kg with various strawberries or quercetin (100 mg/kg) for 10 d (equiv. to 0.5 kg for 70 kg adult)	Reduced MDA, enhanced SOD & in part CAT in gastric mucosa.	Antioxidant enzyme activities increased with strawberry extract, decreased gastric lipid peroxidation. Sign. correlation between total anthocyanin content & % inhibition of ulcer	[87]

Effect of apple PP (APP) on mice with induced colitis	APP at 1% added to drinking water (90% tannins) for up to 4 weeks	APP administration dampened mRNA expression of IL-1*β*, TNF-*α*, IL-6, IL-17, IL-22, CXCL9, CXCL10, CXCL11, & IFN-*γ* in colon	APP-mediated protection required T cells. Giving APP during colitis to T-cell receptor (−/−) mice enhanced proinflammatory cytokine expression, showing need for TCR*αβ* cells in APP-mediated protection	[88]

Effect of ellagic acid (EA) & enriched pomegranate extract (PE) in TBNS induced rats	6 weeks with either 250 or 500 mg/kg PE, or 10 mg/kg EA, or both together	MPO activity & TNF-*α* levels were significantly reduced in rats receiving PP	PE & EA-enriched PE diets decreased COX-2 & iNOS expression, reduced MAPK phosphorylation & prevented NF-*κ*B translocation	[63]

Effect of EGCG & *Piper nigrum* on DSS induced colitis in mice	6.9 mg/kg bw. EGCG or *Piper nigrum* (2.9 mg/kg) for 60 d	Combination of EGCG & piperine sign. reduced loss of bw., improved clinical course, & increased overall survival	Attenuated colitis was associated with reduced histological damage to colon & reduction of tissue concentrations of MDA. Neutrophil accumulation indicator MPO was reduced in the colon; SOD & GPx were increased	[89]

Effect of green tea PP on DSS induced colitis in IL-10 deficient mice	Green tea PP or EGCG at 0.25, 0.5, & 1% added to diet for 10 weeks	Low dose improved histopathology; all doses improved antioxidant levels (colonic & hepatic GSH), reduction of circulating TNF-*α* & IL-6	Antioxidant activities of polyphenols	[58]

Effect of grape juice on rats with TBNS induced colitis	1 or 2% grape juice in diet for up to 9 d	1% grape juice improved clinical symptoms of colitis: reduced intensity of macroscopic & histological scores	Sig. differences of TNF-*α* & inducible NO synthase mRNA expression	[90]

Effect of oligonol (lychee PP) on mice with DSS induced colitis	0.5 or 5 mg/kg/d for 2 weeks	Oligonol sign. inhibited activation of NF-*κ*B, STAT3, COX-2, iNOS & cyclin D1 in the colon. It also inhibited adenoma formation & attenuated MDA levels & protein oxidation (4-hydroxy-2-nonenal)	Various anti-inflammatory genes involved, as well as effects on antioxidant status	[91]

Effects of *Phlomis purpurea *L. & *Phlomis lychnitis *L. on rats with DSS induced colitis	*P. lychnitis* (10 & 20 mg/kg), *P. purpurea* (10 & 25 mg/kg) for 1 week	Both extracts reduced colonic MPO activity, increased colonic GSH, & downregulated iNOS expression. Only *P. purpurea* extract reduced expression of IL-1*β* & IL-17, CINC-1 & MCP-1, & ICAM-1	Anti-inflammatory aspects of both extracts. Implication of NF-*κ*B?	[92]

Effect of green tea PP on Mdr1a(−/−) mice on proteomic & transcriptomic endpoints	0.6% in the diet for 12 weeks	Improved histopathology, reduced abundance of transcripts & proteins associated with immune & inflammatory response/fibrinogenesis, increased abundance of pathways associated with xenobiotic metabolism in response to GrTP	Anti-inflammatory activity mediated by multiple molecular pathways. PPAR-*α* & STAT1 appear to be key molecules regulating these effects	[93]

Effect of naringenin on DSS induced colitis mice	0.3% naringenin in diet for up to 9 d	Naringenin attenuated the increased DAI & colon shortening & suppressed the increased cytokine (IL-17A, IL-1*β*, IL-6, MIP2 expression). Reduction of permeability	Anti-inflammatory properties of naringenin & barrier protection	[94]

Effect of grape seed & marc extract (GSME) on healthy pigs	GSME at 1% added to diet in 6 pigs versus 6 control pigs for 4 weeks	Lower expression of NF-*κ*B (ICAM-1, ccl-2, IL-8, TNF-*α*, SAA) & Nrf-2 (GPx-1, NQO-1, PRDX-6, SOD-1, TXNRD-1) target genes. No difference of conc. of plasma *α*-tocopherol & TBARS in liver & plasma & total antioxidant capacity	Pigs fed GSME diet had lower NF-*κ*B & Nrf-2 transactivation in duodenal mucosa. Ratio of villus height : crypt depth & the gain : feed ratio was higher in pigs fed GSGME	[95]

Effect of catechin on rats receiving ketoprofen	Catechin (35 mg/kg/d) for 21 d	Catechin inhibited oxidative damage & reversed impairment of antioxidant system (GSH, LDH-leakage, 8-OHdG) in intestinal mucosa	ROS reduction by polyphenols	[96]

*Passiflora edulis* peel rich in fiber/PP on TBNS induced colitis in rats	7 d, no PP conc. of passion fruit stated. 25 g passion fruit flour/kg diet given	Improved serum FRAP, GPx, TBARS, GR, decreased colon lipid peroxidation, decreased no. of aerobic bacteria & Enterobacteria, improved acetic & butyric acid levels in feces, higher no. of Bifidobacteria & Lactobacilli	Improved antioxidant status, improved gut flora	[97]

Effect of PP rich evening primrose pomace (EP) extract on TBNS induced colitis in mice	10 mg/kg of 612 mg/g PP extract per dry basis, for 3 d	Improved histopathology & MPO, reduced tissue hydrogen peroxide levels, no effect on IL-1*β*, TNF-*α*	Reduced ROS via antioxidant activity	[98]

PP-rich sorghum bran given to rats with DSS induced colitis	6% fiber diet given over 21 d	Diet significantly affected *Bacteroidales*, *Bacteroides*, *Clostridiales,* & *Lactobacillus*	Protection via improving microbial diversity & richness & dysbiosis of Firmicutes/Bacteroidetes	[99]

Effect of grape seed PP in IL-10 deficient mice	16 weeks of exposure with 1% GSP of dry food weight	Improved histopathology, reduced pore forming claudin-2 protein, & increased barrier forming claudin-1 protein expression	Reduced expression of NF-*κ*B, reduced beclin-1 & AMPK expression by GSE	[100]

Effects of grape seed PP (GSP) in DSS induced colitis in rats	21 d, 1.15 mg/g PP in diet	Reduced lesions (histological score) & disease activity index, reduced cytokines (IL-13, TNF-*α*, IL-1*β*, IL-10, GM-CSF, IL-6, IL-1*α*, IF-*γ*), reduced MPO, enhanced GSH in colonic tissue	Upregulation of various genes implicated in colitis such as intercellular adhesion molecule 1 (ICAM-1) & matrix metalloproteinase 9 (MMP-9)	[101]

Effects of gallic acid on induced colitis in mice	10 mg/kg for 7 d together with DSS	Improved histology scores, reduced TNF-*α*, IL-6, IL-1*β*, IL-17, IFN-*γ* expression in colonic tissue	Reduced expression of p-STAT3, reduced expression of iNOS, COX-2, MPO in colon, reduction of p65-NF-*κ*B	[102]

Effect of wheat anthocyanins on DSS induced colitis in mice	No dose specified. 14 d exposure	No sign. effects on colon length, bw., histopathology, markers of oxidative stress (FRAP, TAC, AOPP)	Degradation of anthocyanins, unclear dose, too much focus on antioxidant effects only	[103]

Effect of cranberry extract on mice	C57BL/6J mice on high fat/high sucrose diet receiving either water or cranberry extract for 8 weeks	Mice receiving cranberry extract showed reduced intestinal oxidative stress & inflammation	Enhanced population with *Akkermansia* (mucus-degrading, SCFA producing), prebiotic effect	[104]

Effect of cacao extract on mice with DSS induced colitis	5 & 10% cacao diets for 62 d	Inhibited proliferation of tumor epithelial cells, suppressed colonic IL-6, TNF-*α*, IL-17, IL-1*β* expression	Reduced expression & activation of STAT3, NF-*κ*B, reduced expression of Bcl-xl, CD68^+^, & MPO, enhanced caspase-3	[105]

See footnote of Table 3.

**Table 5:** In vitro trials suggesting health benefits of polyphenols with respect to IBD—an overview.

Aspects studied	Dosing and time	Effects found	Mechanism proposed and critics	Reference
Effect of phytolens (water-soluble extract of PP antioxidants from nonsoy legumes) on colonic (T84) & murine macrophage (RAW 264.7) cells	Phytolens (10–100 *μ*M/mL) exposed to T84 & RAW 264.7 cells, 4 h & 12 h of exposure	Phytolens sign. attenuated apoptosis in T84 cells induced by ONOO^−^. Phytolens did not directly affect T84 cell viability or induce apoptosis after 4 h or overnight exposure. RAW 264.7 cells exposed to phytolens displayed decreased cell viability & increased apoptosis	Potential beneficial effects of phytolens on inflammation via attenuating induced apoptosis	[106]

PP standards effects of Caco-2 cells	Chrysin, ellagic acid, genistein, & EGCG (all 50 *μ*M) for 28 h	Chrysin & ellagic acid inhibited NF-*κ*B activity & genistein & resveratrol increased it. Mixed effects on IL-8 secretion	Anti- & proinflammatory aspects of PP	[107]

Effect of PP metabolites 3,4-dihydroxyphenyl-acetic acid (ES) & 3-(3,4-dihydroxy-phenyl)-propionic acid (PS) on LT97 colon cells	Cells incubated with ES (0–18 *μ*M) & PS (0–90 *μ*M), metabolites of quercetin & chlorogenic acid/caffeic acid, respectively, up to 72 h	PP metabolites did not affect cell number but sign. upregulated GSTT2 expression & decreased COX-2	Intestinal metabolites showed anti-inflammatory properties	[108]

Effect of red wine PP extract (from Lenoir grapes) on human colon-derived CCD-18 Co myofibroblasts cells	0–100 *μ*g/mL GAE, for 24 h	Red wine extract decreased mRNA expression of NF-*κ*B, ICAM-1, VCAM-1, & PECAM-1, in a dose dependent manner	miR-126, a target region within the 3′-UTR of VCAM-1 mRNA, was increased at 100 *μ*g/mL	[109]

Effect of PP-rich grape seed (GS) & grape marc (GM) on Caco-2 cells	24 h with up to 2 mg/mL	Decreased mRNA levels of NF-*κ*B target genes IL-1*β*, IL-8, MCP-1 & CXCL1 in Caco-2 cells. Unchanged mRNA levels of Nrf-2 target genes GPX-2, NQO1, CYP1A1 & UGT1A1	2 mg/mL ethanolic extracts dose-dependently reduced NF-*κ*B transactivation. No effect of ethanolic extracts was observed on Nrf-2	[110]

Effect of apple peel PP on Caco-2/15 cells	250 *μ*g/mL apple peel PP for 24 h	Apple peel PP prevented Fe/ascorbate-mediated lipid peroxidation & counteracted LPS-mediated inflammation by downregulating cytokines (TNF-*α*, IL-6) & PGE2	Downregulation of COX-2 & NF-*κ*B. Also induction of Nrf-2	[111]

Effect of red wine extract (RWE) (rich in catechin B1 & malvidin-3-glucoside) on HT-29 colon cells	100–600 *μ*g/mL RWE extract (144 mg/g PP) for 24 h	RWE suppressed I*κ*B degradation & IL-8 production dose-dependently. It also inhibited increase of NO from iNOS & of protein tyrosine nitration (biomarker of nitrosative stress)	RWE reduced NF-*κ*B activation; COX-2 & iNOS	[69]

Effect of catechin, theaflavin, malvidin, cyanidin & apigenin on human Int-407 cells treated with ketoprofen	25 *μ*M (malvidin, cyanidin & apigenin) or 100 *μ*M (catechin & theaflavin) for 5 h	Catechin sign. decreased levels of lipid peroxidation & ROS (MDA, DCF) & increased activity of intracellular antioxidant enzymes GPx, Gred, total sulfhydryl groups (TSH)	ROS reduction of PP. WB analysis revealed that catechin stimulated a time-dependent increase in Nrf-2 & total HO-1 protein expression	[96]

Effect of cyanidin-3-glucoside on HT-29 colon cells	25 *μ*M cyanidin-3-glucoside, up to 24 h	Improved NO, PGE2 & IL-8 production	Improved iNOS & COX-2 expressions. No effect on NF-*κ*B or p38 MAPK, but STAT1	[112]

Effect of blueberry anthocyanin-rich extract on Caco-2 cells	Up to 100 *μ*g/mL up to 48 h	Reduced IL-1*β* levels in Caco-2 cells	Reduced activation of NF-*κ*B. 50 & 100 *μ*g mL^−1^ effective	[113]

Effect of resveratrol on HT-29 colon cells	25 *μ*M resveratrol, up to 24 h	Decreasing levels of activated STAT1 in nucleus. Also reduction of cytokine-stimulated activation of SAPK/JNK pathway	Downregulation of JAK-STAT pathway, though not counteracting cytokine-triggered negative feedback mechanism of STAT1 through p38 MAPK	[114]

Plum & cabbage digesta: triple culture cell model: Caco-2/HT-29-MTX with THP-1	18 h of incubation following digestion	Reduction of IL-8 secretion by plum varieties	Influences via NF-*κ*B & Nrf-2	[67]

See footnote of Table 3.
